# Transcriptome Analysis Reveals the Neuro-Immune Interactions in Duck Tembusu Virus-Infected Brain

**DOI:** 10.3390/ijms21072402

**Published:** 2020-03-31

**Authors:** Junqin Zhang, Yunzhen Huang, Linlin Li, Jiawen Dong, Ming Liao, Minhua Sun

**Affiliations:** Guangdong Provincial Key Laboratory of Livestock Disease Prevention, Scientific Observation and Experiment Station of Veterinary Drugs and Diagnostic Techniques of Guangdong Province, Ministry of Agriculture, Guangdong Open Laboratory of Veterinary Public Health, Institute of Animal Health, Guangdong Academy of Agricultural Sciences, Guangzhou 510640, Guangdong, China; junqinzhang@yeah.net (J.Z.); huangyunzhen110@hotmail.com (Y.H.); lingdang1000@163.com (L.L.); hellendongjiawen@163.com (J.D.)

**Keywords:** Transcriptome analysis, DTMUV, brain, molecular mechanism, neuro-immune interaction, host–pathogen interaction

## Abstract

The duck Tembusu virus (DTMUV) is a mosquito-borne flavivirus. It causes severe symptoms of egg-drop, as well as neurological symptoms and brain damage in ducks. However, the specific molecular mechanisms of DTMUV-induced neurovirulence and host responses in the brain remain obscure. To better understand the host–pathogen and neuro-immune interactions of DTMUV infection, we conducted high-throughput RNA-sequencing to reveal the transcriptome profiles of DTMUV-infected duck brain. Totals of 117, 212, and 150 differentially expressed genes (DEGs) were identified at 12, 24, and 48 h post infection (hpi). Gene ontology (GO) and Kyoto Encyclopedia of Genes and Genomes (KEGG) enrichment analyses uncovered genes and pathways related to the nervous system and immune responses in duck brain. Neuro-related genes, including WNT3A, GATA3, and CHRNA6, were found to be significantly downregulated. RIG-I-like receptors (DHX58, IFIH1) and Toll-like receptors (TLR2 and TLR3) were activated, inducing the expression of 22 interferon stimulated genes (ISGs) and antigen-processing and -presenting genes (TAP1 and TAP2) in the brain. Our research provides comprehensive information for the molecular mechanisms of neuro-immune and host–pathogen interactions of DTMUV.

## 1. Introduction

Duck Tembusu virus (DTMUV) is a newly emerging avian pathogenic mosquito-borne Flavivirus. This virus was first identified as the causative agent of duck egg-drop syndrome in China in 2010 [1]. It infects a variety of duck breeds, resulting in egg drop, anorexia, ataxia, paralysis, and even death with a mortality ranging from 5% to 30% [2,3]. It causes tremendous economic losses in the poultry industry in China and Southeast Asia [4]. In addition, it is capable of infecting and causing cytopathic effects in various mammalian cell lines [5]. Moreover, in one study, DTMUV-specific antibodies and viral RNA were detected in duck farm workers [6]. Convincing evidence has indicated that DTMUV not only poses a great threat to the poultry industry but is also a potential public health hazard.

Tembusu virus (TMUV) is a mosquito-borne Ntaya subgroup flavivirus. It was first identified in *Culex tritaeniorhynchus* in 1955, and has been isolated from *Culex* mosquitoes in the 1970s in Malaysia [7]. A TMUV field strain has also been isolated from *Culex* mosquitoes in China, and a phylogentic analysis demonstrated that it is closely related to DTMUV [8]. Sanisuriwong et al. collected 30,841 mosquitoes from different duck farms in Thailand. They classified and pooled the mosquitoes together, and one pool of *Culex tritaeniorhynchus* was positive for DTMUV. A phylogenetic analysis revealed that this mosquito-derived DTMUV was most closely related to the 2013 Thai DTMUVs [9]. However, at present, no direct evidence has shown that mosquitoes transmit DTMUV to humans and duck flocks. Overall, the *Culex* mosquito and ducks have been implicated as a vector and a reservoir for DTMUV, respectively [10]. Although DTMUV has not been documented to cause human diseases, the potential risk for a virulent DTMUV strain infecting humans still exists.

Many mosquito-borne flaviviruses, including the West Nile virus (WNV), Usutu virus (USUV), Japanese encephalitis virus (JEV), and Zika virus, are capable of causing severe neurological illness [11,12,13,14]. Similar to these flaviviruses, DTMUV is also neuroinvasive and neurovirulent, and can cause neurological symptoms. It was documented that some DTMUV-infected laying ducks exhibited an uncoordinated gait, and were reluctant or unable to walk [1]. Histological examination showed that focal gliosis occurred occasionally in the brain, with lymphocyte infiltration under cranial arachnoid [1]. DTMUV can also result in neurological signs in 20-day-old ducklings [15]. In Malaysia, 2-week-old Pekin ducks inoculated with Tembusu virus displayed histological lesions in the brain and spinal cord [16]. It has been reported that age has an effect on the pathogenesis of Tembusu virus in the Cherry Valley duck. Younger (5-day-old) ducklings developed much more severe neurological dysfunction and higher viral copy numbers compared with older (2-week-old or 5-week-old) ducks after DTMUV infection [17]. Another study demonstrated the same scenario, with all infected ducks (1, 3, and 7 week of age) displaying viral encephalitis [18]. Furthermore, the duck Tembusu virus exhibits neurovirulence and age-dependent neuroinvasiveness in BALB/c mice [5,19]. However, the interaction between DTMUV neurovirulence and neuro-immune responses remains unknown.

In order to reveal the interaction between DTMUV neurovirulence and neuro-immune responses, we used DTMUV to infect 10-day-old specific pathogen free (SPF) ducks in the current study and harvested the duck brains at 12, 24, and 48 h post infection (hpi). The DTMUV-infected and uninfected duck brains were subjected to high-throughput RNA-sequencing. Genes and pathways related to nervous system and immune responses in duck brain were uncovered through gene ontology (GO) and Kyoto Encyclopedia of Genes and Genomes (KEGG) enrichment analyses. Our research reveals the transcriptome profiles of DTMUV-infected duck brain, providing valuable information for the molecular mechanisms of neuro-immune interactions in the brain.

## 2. Results

### 2.1. Detection of Viral Infections in the Brain and Other Organs

**Figure 1:**
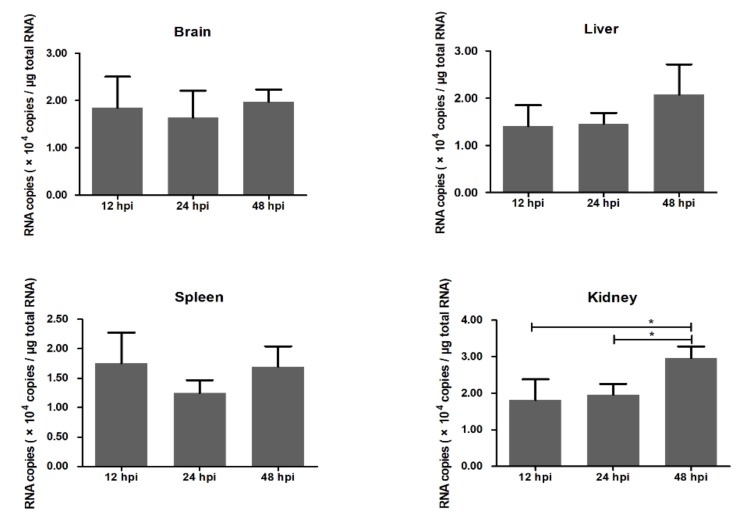
Duck Tembusu virus (DTMUV) RNA copies in the brain, liver, spleen, and kidney. DTMUV RNA copies were detected by absolute quantitative real-time PCR. Y-axis represents RNA copies in one microgram total RNA. * means *p* ≤ 0.05.

In order to detect the viral infection in ducks, we use absolute quantitative real-time PCR to detect the DTMUV RNA copies in the brain, liver, spleen, and kidney (Figure 1). It turns out that the viral RNA is detectable in these four organs at 12, 24, and 48 hpi. The average DTMUV RNA copies in the brain are 1.8451 × 10^4^ at 12 hpi, 1.6435 × 10^4^ at 24 hpi, 1.9728 × 10^4^ at 48 hpi in every microgram total tissue RNA. No signification variation in viral RNA is recorded at the three time points in the brain.

### 2.2. Identification of Differentially Expressed Genes (DEGs)

**Figure 2:**
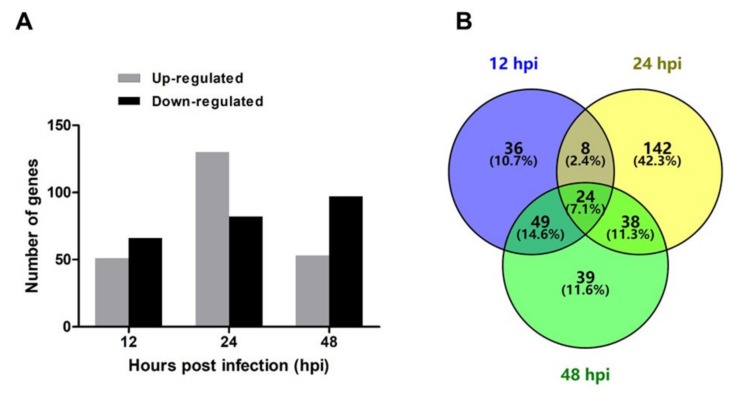
Differentially expressed genes (DEGs) identified at 12, 24, and 48 hpi. (**A**) Numbers of upregulated and downregulated genes at 12, 24, and 48 hpi. (**B**) Venn diagram of DEGs at 12, 24, 48 hpi. The image shows the numbers of common and unique DEGs.

**Figure 3:**
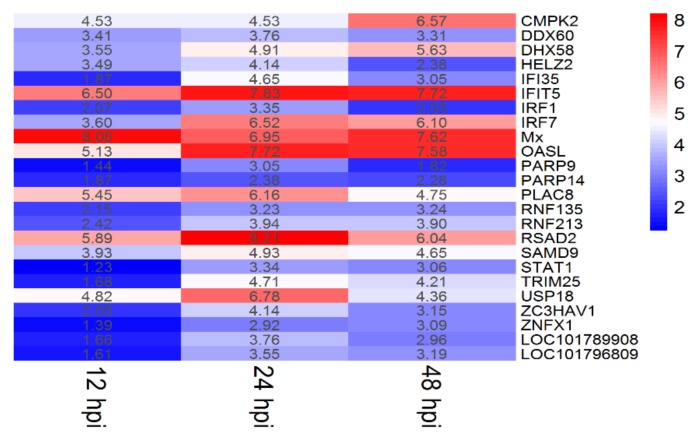
Twenty-four common genes shared by 12, 24, and 48 hpi. The number represents log_2_ (fold change) of each gene at different time points.

To study the transcriptome profile of DTMUV-infected duck brains, six sequencing libraries were prepared at 12, 24, and 48 hpi. A total of 304,564,722 raw reads were produced from the six libraries. The number of clean reads ranged from 40,030,410 to 57,108,946 after filtering out ribosomal RNA, adapters, and low quality reads (Table 1). The raw sequencing data have been deposited in the NCBI Gene Expression Omnibus (GEO) under the accession number GSE142592. We identified 479 DEGs under the criteria of *p*-value ≤ 0.05 and ｜log_2_ (fold change)｜ ≥ 1 at the three time points. Among the 479 DEGs, 117, 212, and 150 genes were identified at 12, 24, and 48 hpi, respectively. At 12 hpi, 51 DEGs were upregulated and 66 DEGs were downregulated; at 24 hpi, 130 DEGs were upregulated and 82 DEGs were downregulated; and at 48 hpi, 53 DEGs were upregulated and 97 DEGs were downregulated (Figure 2A). A Venn diagram revealed that there were 24 common DEGs at three tested time points (Figure 2B). The specific information and fold-changes of the 24 DEGs were displayed in Figure 3. All the 24 DEGs were significantly upregulated. Meanwhile, the 24 DEGs were mainly interferon stimulated genes (ISGs), including CMPK2, DDX60, DHX58, HELZ2, IFI35, IFIT5, IRF1, IRF7, Mx, OASL, PARP9, PARP14, PLAC8, RNF135, RNF213, RSAD2, SAMD9, STAT1, TRIM25, USP18, ZC3HAV1, and ZNFX1. Among these ISGs, RSAD2, IFIT5, and OASL were upregulated more than 200-fold.

### 2.3. GO and KEGG Enrichment Analysis

**Figure 4:**
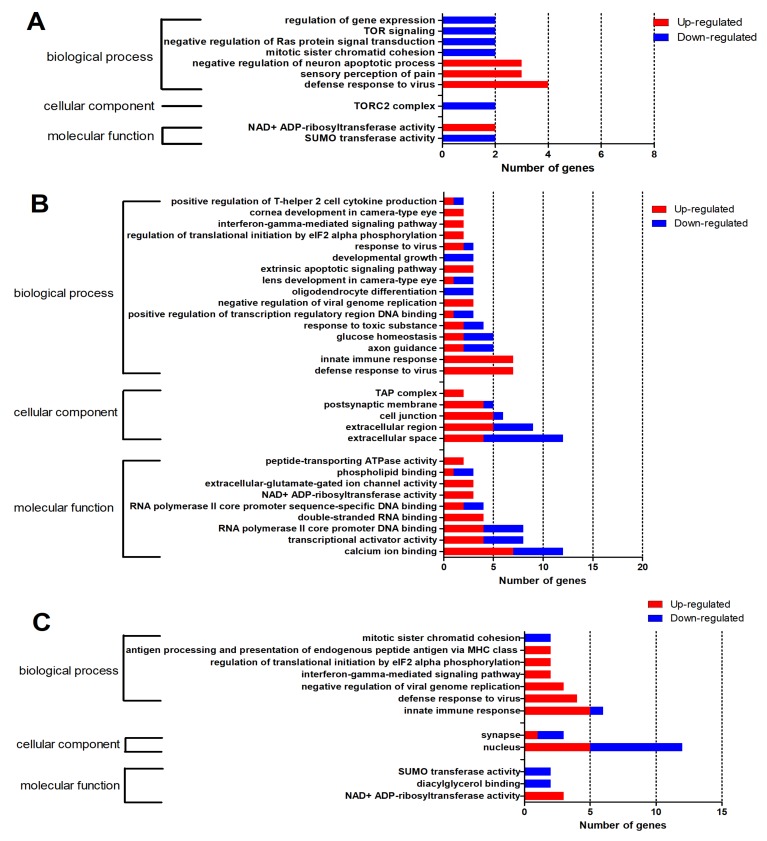
Gene ontology (GO) enrichment analysis of DEGs (*p* < 0.05). (**A**) Ten GO terms significantly enriched at 12 hpi. (**B**) Thirty GO terms significantly enriched at 24 hpi. (**C**) Twelve GO terms significantly enriched at 48 hpi. The red portion of the bar represents the number of upregulated genes, and the blue portion of the bar represents the number of downregulated genes.

To address the gene functions, the 479 DEGs were subjected to DAVID for a GO enrichment analysis. As shown in Figure 4, a total of 52 GO terms (*p* < 0.05) were classified into three main GO categories, including biological process (BP), cellular component (CC), and mechanism functions (MF). Among the 52 GO terms, 10 GO terms were enriched at 12 hpi, including seven in the BP category, one in the CC category, and two in the MF category (Figure 4A); 30 GO terms were enriched at 24 hpi, including 16 in the BP category, five in the CC category, and nine in the MF category (Figure 4B); and 12 GO terms were enriched at 48 hpi, including seven in the BP category, two in the CC category, and three in the MF category (Figure 4C). Two GO terms—defense response to virus and NAD + ADP-ribosyltransferase activity—were enriched at all three time points. The specific information of GO terms is listed in Table S1.

**Figure 5:**
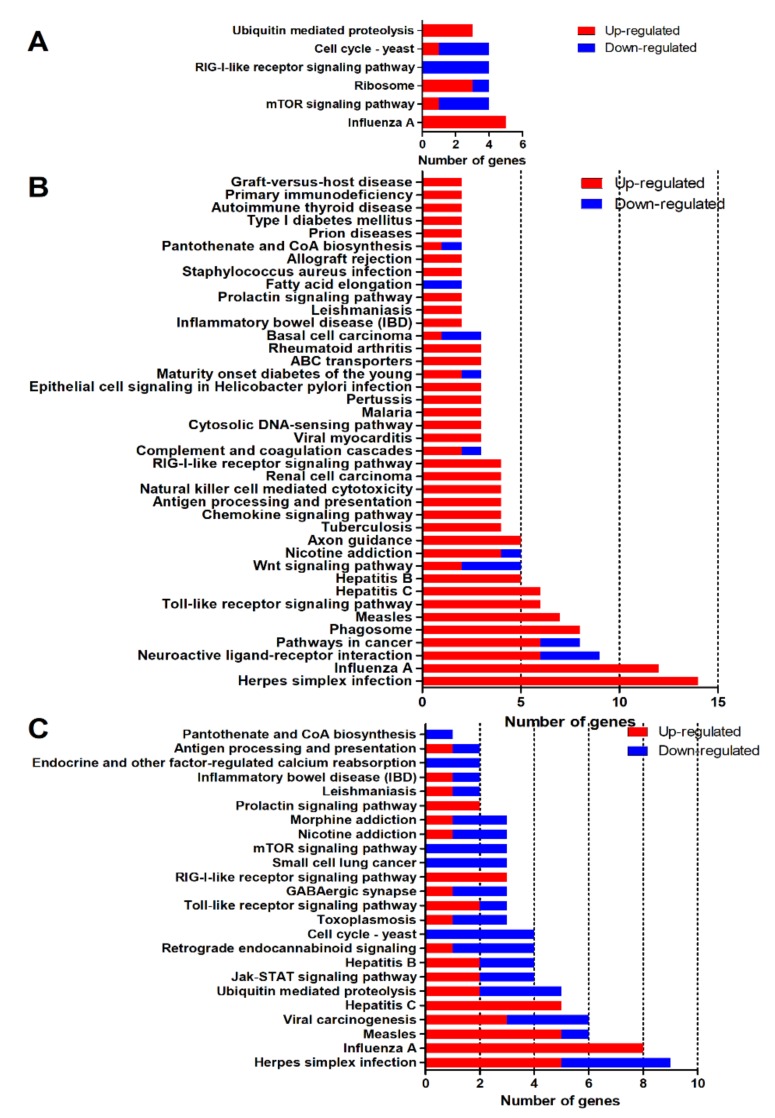
Kyoto Encyclopedia of Genes and Genomes (KEGG) enrichment analysis of DEGs (*p* < 0.05). (**A**) Six KEGG terms were significantly enriched at 12 hpi. (**B**) Forty KEGG terms were significantly enriched at 24 hpi. (**C**) Twenty-four KEGG terms were significantly enriched at 48 hpi. The red portion of the bar represents the number of upregulated genes, and the blue portion of the bar represents the number of downregulated genes.

To explore the biological processes and molecular function during DTMUV infection, a KEGG enrichment analysis was performed. As shown in Figure 5, a total of 70 KEGG pathways (*p* < 0.05) were identified during the process of DTMUV infection. There were 6, 40, and 24 KEGG pathways enriched at 12 (Figure 5A), 24 (Figure 5B), and 48 hpi (Figure 5C), respectively. The pathways were mainly related to pathogen infection, immune response, and the nervous system. Two common pathways, Influenza A and the RIG-I-like receptor signaling pathway, were enriched at all three time points. The details of DEGs that were enriched in different pathways are shown in Table S2.

### 2.4. Pathways Related to Nervous System

**Figure 6:**
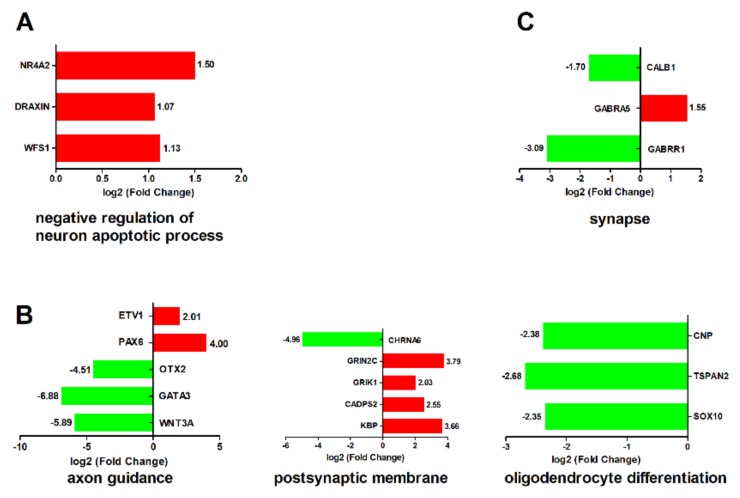
GO terms related to the nervous system (*p* < 0.05). (**A**) Negative regulation of neuron apoptotic process was significantly enriched at 12 hpi. (**B**) Axon guidance, postsynaptic membrane, and oligodendrocyte differentiation were significantly enriched at 24 hpi. (**C**) Synapse was significantly enriched at 48 hpi. The X-axis represents log_2_ (fold change), and the Y-axis represents genes enriched in each GO term. The red bar represents the upregulated gene, and the green bar represents the downregulated gene.

**Figure 7:**
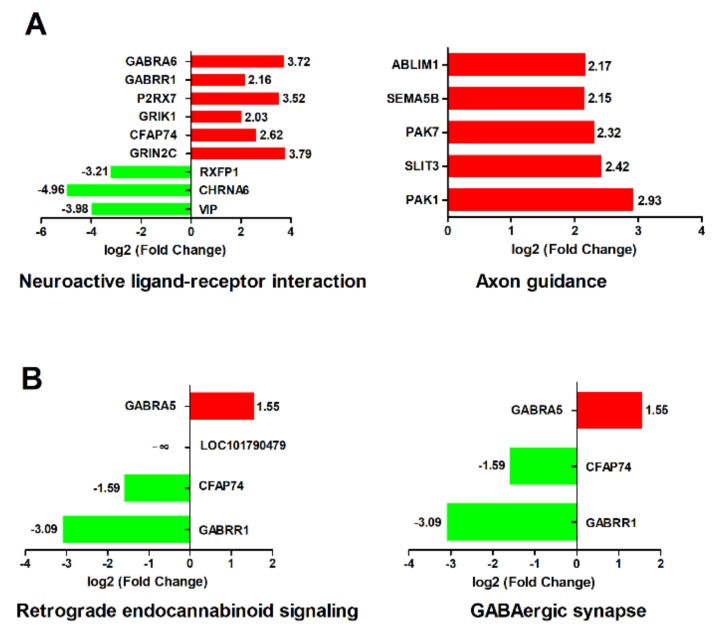
KEGG terms related to nervous system (*p* < 0.05). (**A**) Neuroactive ligand–receptor interaction and the axon guidance pathway were significantly enriched at 24 hpi. (**B**) Retrograde endocannabinoid signaling and the GABAergic synapse pathway were significantly enriched at 48 hpi. The X-axis represents log_2_ (fold change), and the Y-axis represents genes enriched in each KEGG pathway. The red bar represents the upregulated gene, and the green bar represents the downregulated gene.

DTMUV infection causes neurological symptoms of anorexia, ataxia, and paralysis in ducklings [3,15]. To elucidate the biological processes and pathways related to the nervous system after DTMUV infection, we classified the GO terms (Figure 6) and KEGG pathways (Figure 7) relevant to the nervous system (*p* < 0.05). GO analysis revealed that five terms were significantly enriched, including negative regulation of neuron apoptotic process, axon guidance, postsynaptic membrane, oligodendrocyte differentiation, and synapse categories (Figure 6). Negative regulation of neuron apoptotic process pathway was significantly enriched at 12 hpi (Figure 6A); axon guidance, postsynaptic membrane, and oligodendrocyte differentiation pathway were significantly enriched at 24 hpi (Figure 6B); and the synapse category pathway was significantly enriched at 48 hpi (Figure 6C). In the axon guidance category, genes like WNT3A, GATA3, and OTX2 were significantly downregulated by about 59.30-, 117.78-, and 22.78-fold. In the oligodendrocyte differentiation category, SOX10, TSPAN2, and CNP were all significantly downregulated. 

A KEGG analysis revealed that four pathways, including neuroactive ligand-receptor interaction, axon guidance, retrograde endocannabinoid signaling, and GABAergic synapse pathways, were significantly enriched (Figure 7). Neuroactive ligand–receptor interaction and the axon guidance pathway were significantly enriched at 24 hpi (Figure 7A). Retrograde endocannabinoid signaling and the GABAergic synapse pathway were significantly enriched at 48 hpi (Figure 7B). In the neuroactive ligand–receptor interaction pathway, six genes were significantly upregulated and three genes were significantly downregulated. Among these six upregulated genes, GRIN2C, P2RX7, GABRR1, and GABRA6 were neurotransmitters. The three downregulated genes were RXFP1, CHRNA6, and VIP. However, the acetylcholine receptor, CHRNA6, was significantly downregulated by 31.12-fold. In the axon guidance pathway, all five genes (ABLIM1, SEMA5B, PAK7, SLIT3, and PAK1) were significantly upregulated. In the retrograde endocannabinoid signaling and the GABAergic synapse pathway, only GABRA5 was significantly upregulated; CFAP74 and GABRR1 were significantly downregulated.

### 2.5. Pathways Related to Immune Responses

Host immune responses play pivotal roles during viral infection. In our study, seven immune-related KEGG pathways were involved in the process of DTMUV infection, including the RIG-I-like receptor signaling pathway, the Toll-like receptor signaling pathway, the JAK-STAT signaling pathway, the chemokine signaling pathway, antigen processing and presentation, natural killer cell mediated cytotoxicity, and complement and coagulation cascades (*p* < 0.05) (Table 2). Among these seven pathways, the antigen processing and presentation pathway belongs to the adaptive immune response. The other six pathways belong to the innate immune response.

The innate immune response is the first line of a host’s anti-viral response. As shown in Table 2, the RIG-I-like receptor signaling pathway is activated at 12 hpi, 24 hpi, and 48 hpi. DHX58 (also known as LGP2), IRF7, and TRIM25 in the RIG-I-like receptor signaling pathway were significantly upregulated at all three time points. In addition, the RIG-I-like receptor (RLR) family member IFIH1 (also known as MDA5) was significantly upregulated at 24 hpi. The Toll-like receptor signaling pathway was significantly enriched at 24 hpi and 48 hpi. TLR2 was significantly upregulated at 24 hpi. Nevertheless, TLR2 was significantly downregulated at 48 hpi. In addition, TLR3 was also significantly upregulated at 24 hpi. 

The adaptive immune response helps to eliminate pathogens. Antigen processing and presentation is essential for the adaptive immune response. In the antigen processing and presentation pathway, transporter-associated with antigen processing 1 (TAP1) and transporter-associated with antigen processing 2 (TAP2) were significantly upregulated at 24 hpi. However, at 48 hpi, only beta2-microglobulin (B2M) was significantly upregulated. 

### 2.6. Validation of RNA-seq by Quantitative Real-Time Polymerase Chain Reaction (qRT-PCR)

**Figure 8:**
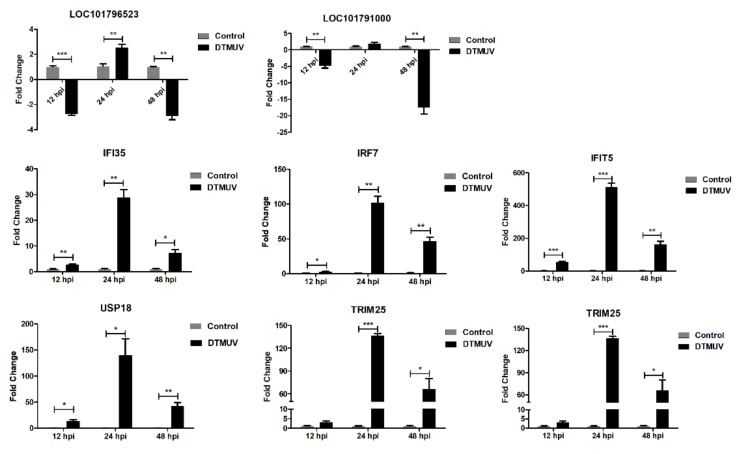
Validation of RNA-seq by qRT-PCR. The X-axis represents hours post infection (hpi). The Y-axis represents fold change. * means *p* ≤ 0.05, ** means *p* ≤ 0.01, *** means *p* ≤ 0.001.

To verify the reliability of RNA-seq, a qRT-PCR analysis was performed. Eight genes, including LOC101796523, LOC101791000, IFI35, IRF7, IFIT5, USP18, TRIM25, and RSAD2, were randomly selected for qRT-PCR analysis (Figure 8). Although the exact fold changes were not completely identical, the expression patterns of these eight genes obtained from qRT-PCR and RNA-seq were matched. Thus, the RNA-seq dataset was reliable.

## 3. Discussion

Flaviviruses are hazardous to both humans and animals. Although duck Tembusu virus is an avian mosquito-borne flavivirus, its detection and isolation in humans and other non-avian species has revealed its zoonotic potential. Moreover, the duck Tembusu virus displays neurovirulence in ducks, goslings, and mice [5,17,18,19,20]. Nevertheless, there is insufficient knowledge on the molecular mechanisms of DTMUV infection in the brain. The current study reveals a panorama of host–pathogen and neuro-immune interactions.

### 3.1. Genes Related to Nervous System

In our research, DTMUV-infected ducks exhibited depression and neurological symptom of ataxia. Many nervous system-related genes were enriched, as demonstrated by the GO terms and KEGG pathways. Among these genes, WNT3A, GATA3, and CHRNA6 were the top three significantly downregulated genes. WNT3A is a Wnt ligand, activating the Wnt signaling pathway [21]. It has been reported that WNT3A is critical for the development and function of the nervous system [22], as well as for glucose metabolism in cortical neurons [23]. Thus, DTMUV may affect the development of nervous system through inhibition of WNT3A. GATA3 is a transcription factor that plays an essential role in sympathetic neuron development [24,25,26]. The sympathetic nervous system innervates nearly every tissue in the body and keeps the body at a state of raised activity and attention [27,28]. The inhibition of GATA3 caused the inhibition of sympathetic neuron development in the brain after DTMUV infection. Moreover, the sympathetic nervous system also modulates the immune system and inflammation [29]. There may be an implied relationship between host–pathogen and neuro-immune interactions through the inhibition of GATA3. Cholinergic receptor nicotinic alpha 6 (CHRNA6) is a subunit of neuronal nicotinic acetylcholine receptors that mediate neurotransmission [30,31]. The significant decrease of CHRNA6 indicated the inhibition of neuronal nicotinic acetylcholine receptor genesis. Consequently, the downregulation of WNT3A, GAGA3, and CHRNA6 may account for the DTMUV-induced depression and neurological symptoms. 

### 3.2. Immune Responses in Duck Brain

In our research, both the innate immune response and the adaptive immune response were suppressed in duck brain tissues during the process of DTMUV infection. The innate immune response provides an initial barrier against pathogens. Pattern recognition receptors (PRRs) recognize the pathogen-associated molecular patterns (PAMPs) of the virus. PRRs, including DHX58 (LGP2), IFIH1 (MDA5), TLR2, and TLR3, were involved in DTMUV infection in our research. DHX58 and IFIT1 belong to the RIG-I-like receptors (RLRs), which are activated by sensing viral RNAs in the cytosol [32]. Another important member in the RLRs family is RIG-I, which has been reported to be upregulated in the brain of DTMUV-infected five-day-old Cherry Valley ducks [33]. However, RIG-I was not activated during experimental infection of ducks in our research. We speculate that the expression differences of RIG-I in these two studies may be attributed to the use of different duck breeds, virus strains and virus dosages. In our study, DHX58 (LGP2) was activated at 12, 24, and 48 hpi; while IFIH1 (MDA5) was activated only at 24 hpi. The relationship between LGP2 and MDA5 remains controversial [34]. It has been reported that LGP2 and MDA5 work together in antiviral signal transduction in mammalian cells [35,36]. MDA5 restricts duck enteritis virus (DEV) replication and LGP2 plays a biphasic role in MDA5-mediated anti-DEV event [37]. Thus, the stimulation of MDA5 during DTMUV infection indicates its antiviral roles in the brain. The exact relationship between duck MDA5 and LGP2 in the brain requires further exploration.

TLR2 and TLR3 belong to the Toll-like receptors (TLRs) Family. TLR3, which is responsible for the recognition of double-stranded RNA (dsRNA), plays a crucial role in various viral infections of the nervous system [38]. Studies have demonstrated that TLR3 mediates flavivirus infection in the nervous system, in case of WNV and JEV infections [39,40]. In the case of WNV infection, the viral load in the brain and the mortality were higher in TLR3-knockout mice compared with wild type mice [40]. TLR3-knockout mice were more susceptible to JEV and induced more severe central nervous system (CNS) inflammation compared with wild-type mice [41]. Li and collaborators have reported the upregulation of TLR3 in the brains of DTMUV-infected ducks [33]. Consequently, we speculate that the increase of TLR3 is associated with the host’s anti-DTMUV effect in the brain. Intriguingly, TLR2 was upregulated at 24 hpi, but downregulated at 48hpi. Since TLR2 is responsible for the recognition of di- and tri-acylated lipopeptides, the upregulation of TLR2 in our study may be related to the recognition of viral proteins. However, the mechanism of TLR2 downregulation remains unconfirmed and requires deep analysis. 

Antigen processing and presentation initiates the adaptive immune response. In the antigen processing and presentation pathway, TAP1, TAP2, and B2M were significantly upregulated. Transporter-associated with antigen processing (TAP) is a heterodimer composed of TAP1 and TAP2. It is located in the membrane of the endoplasmic reticulum (ER), transporting endogenous peptides into the ER lumen. Studies have proven that TAP mediates peptides loading onto the major histocompatibility complex class I (MHC-I) cell surface, resulting in viral evasion of natural killer cell recognition during flavivirus infection [42,43]. However, MHC-I was not detected in the brain in our research. The upregulation of TAP1 and TAP2 in the infected duck brain indicates the activation of the adaptive immune system. B2M, a highly conserved immune molecule in vertebrates, is associated with multiple diseases [44]. Its accumulation in aging blood results in impairments of cognition and neurogenesis [45]. The elevation of B2M in our study hints at the impairment of neurogenesis in duck brain.

### 3.3. ISGs Response to DTMUV Infection in Duck Brain

Upon viral infection, PRRs recognize the PAMPs, stimulating the expression of interferons (IFNs). In turn, IFN signaling leads to the expression of hundreds of ISGs. ISGs play important roles in cells’ intrinsic antiviral defense, antiproliferative activities, and stimulation of adaptive immunity [46]. The interferon (IFN) system is part of the innate immune response. 

In the present study, 22 out of 24 common DEGs were upregulated ISGs. Among the 22 ISGs, IFIT5, Mx, OASL, RSAD2 (also known as viperin), and ZC3HAV1 have already been identified as exerting antiviral effects in mammals [47]. The ISGs may exert a dominant role in anti-DTMUV activity in the brain. Interestingly, IFIT5, OASL, and RSAD2 were upregulated more than 200-fold for at least one time point in our study. IFIT5 is the sole member in the interferon-induced proteins with tetratricopeptide repeats (IFITs) family found in birds, which has been demonstrated as playing an important role in the immune response to duck hepatitis A virus type 3 (DHAV-3) [48,49]. However, the exact role of IFIT5 in DTMUV infection has not been elucidated. Bi and his colleagues cloned duck OASL from Cherry Valley ducks and characterized its antiviral effects against DTMUV. They found that duck OASL overexpression slightly inhibited DTMUV replication whereas, the knockdown of duck OASL increased DTMUV replication in DF-1 cells [50]. RSAD2 (viperin) is a radical S-adenosyl methionine (SAM) enzyme that plays a multifaceted role in antiviral response [51]. It has been shown to inhibit several flaviviruses [52]. Duck viperin was found to inhibit the budding of DTMUV in BHK-21 cells [53]. Consequently, the highly expressed IFIT5, OASL, and RSAD2 in brain tissues in our study indicate that these genes play pivotal role in defensing against DTMUV in the nervous system. However, the specific molecular mechanisms of these ISGs in anti-DTMUV immune regulation need to be further explored. Meanwhile, genes involved in IFN suppression in mammals, including IFI35, PARP14, and USP18 were also upregulated in our study. The induction of these negative IFN regulators is necessary for the host to avoid excessive IFN-induced immune response. 

## 4. Materials and Methods

### 4.1. Virus and Cells

The Tembusu virus JM strain (GenBank accession number: JN811559.1) was isolated from diseased layer ducks in Guangdong Province, Southern China, in 2011 [54]. Duck embryo fibroblasts (DEFs) were obtained from 9-day-old specific pathogen free (SPF) embryos (purchased from Harbin Veterinary Research Institute, Harbin, China). The virus was propagated in DEFs maintained in DMEM medium supplemented with 10% fetal bovine serum (FBS) (Gibco, Australia), 100 U/mL penicillin, and 0.1 mg/mL streptomycin. The harvested virus was subjected to ultracentrifugation. The purified virus was then resuspended in PBS. The virus titer was 10^5.5^ TCID50/mL.

### 4.2. Animal Experiment

Twenty-four 10-day-old SPF ducks (Harbin Veterinary Research Institute, Harbin, China) were randomly divided into two groups. The ducks were raised in separated negative-pressure isolators. The challenge group was intramuscularly inoculated with 0.5 mL of 10^5.5^ TCID50/mL DTMUV JM strain. The control group was inoculated with an equal amount of sterile PBS. At 12, 24, and 48 hpi, four ducks were randomly selected from each group for euthanasia. The brain, liver, spleen, and kidney were collected and stored in liquid nitrogen. The brain tissues were collected for next generation sequencing (NGS). The animal experiment protocol used in this study was approved by and performed under the guidance of the Committee on the Ethics of Animal Experiments of Institute of Animal Health, Guangdong Academy of Agricultural Sciences Experimental Animal Welfare Ethics Committee on 12 February, 2018 (Approve ID: 2018-010). All efforts were made to minimize animal suffering.

### 4.3. Transcriptome cDNA Library Construction and Sequencing

Total RNA of each sample was extracted and purified using RNeasy Mini Kit (Qiagen, Hilden, Germany) and RNase-Free DNase Set (QIAGEN, Hilden, Germany). RNA concentration was determined by NanoDrop (Thermo Fisher Scientific Inc., USA). RNA was checked for RNA Integrity Number (RIN) by an Agilent Bioanalyzer 2100 (Agilent technologies, USA). RNA samples with RIN ≥ 7 were used for cDNA library construction. Equal quantities of total RNA from each group of four animals were mixed prior to Illumina cDNA library preparation. A next-generation sequencing library was constructed using NEBNext® Ultra™ RNA Library Prep Kit for Illumina (NEB, Ipswich, USA) according to the manufacturer’s protocol. Briefly, the poly(A) mRNA was purified from total RNA using NEBNext Poly(A) mRNA Magnetic Isolation Module (NEB, Ipswich, USA). The mRNA fragmentation was performed using NEBNext First Strand Synthesis Reaction Buffer. First-strand cDNA was synthesized using a random hexamer primer and ProtoScript II Reverse Transcriptase (NEB, Ipswich, USA). Second-strand cDNA was synthesized using Second Strand Synthesis Enzyme Mix. The product was purified using a QIAquick PCR purification kit (Qiagen, Hilden, Germany). The purified double-stranded cDNA was then treated with End Prep Enzyme Mix (NEB, Ipswich, USA) to repair both ends. The repaired fragments were ligated to sequencing adapters by T-A ligation. The products were then purified and enriched by PCR to create the final cDNA library. Sequencing was performed using an Illumina HiSeq 2000 (Illumina, USA). Library construction and sequencing were performed by GENEWIZ Biotechnology Corporation (Suzhou, China).

### 4.4. Transcriptome Data Analysis 

In order to remove technical sequences (including sequencing adapters, polymerase chain reaction (PCR) primers, ribosome RNA, and low-quality reads), raw data in the fasta format were processed by Trimmomatic software (version 0.30) to obtain high-quality clean data. The clean data were aligned and mapped to the reference genome (NCBI Genome Assembly: GCA_000355885.1) by using Hisat2 software (version 2.0.14) [55]. The values of total mapped reads in different samples were computed by HTSeq software (version 0.6.1). Finally, differences in gene expression between mock- and DTMUV-infected groups were calculated and normalized by the fragments per kilobase of the exon model per million mapped reads (FPKM) method [56].

### 4.5. Differential Expression Analysis

A differential expression analysis was conducted using DESeq software (version 1.18.0) [56,57]. DEGs in different groups at 12, 24, and 48 hpi were selected using edgeR (version 3.4.6) [58] by the thresholds of false discovery rate (FDR), adjusted *p*-value ≤ 0.05, and ｜log_2_ (fold change)｜ ≥ 1. The gene ontology (GO) enrichment analysis was performed using DAVID online analysis software (https://david.ncifcrf.gov/tools.jsp). GO terms with corrected *p*-values < 0.05 were considered significantly enriched. The Kyoto Encyclopedia of Genes and Genomes (KEGG) pathway enrichment analysis was also performed online (https://www.kegg.jp) and KEGG pathways with *p*-values < 0.05 were considered statistically significant.

### 4.6. qRT-PCR 

Total RNA of brains, livers, spleens, and kidneys were isolated using TRIzol (Invitrogen, Waltham, USA). The cDNA was obtained using avian myeloblastosis virus (AMV) reverse transcriptase (Takara, Dalian, China) following the manufacturer’s instructions. A quantitative real-time PCR analysis was carried out using SYBR Green master mix (Roche, USA). Primers used in this study were synthesized by Sangon Biotech (Guangzhou, China) (Table S3). The amplification parameters were set as 50 °C for 2 min and 95 °C for 10 min, followed by 40 cycles of 95 °C for 15 s, 60 °C for 30 s, and 72 °C for 30 s. Each sample had four replicates. The absolute quantitative real-time PCR was conducted according to Liu and colleagues’ report [59] with small modifications. One microgram total RNA was taken from each sample to synthesize cDNA in 20 microliter volume. Then one microliter was taken to conduct real-time PCR. A plasmid containing the DTMUV envelope gene (E gene) was used to construct standard curve.

As for the validation of RNA-seq by relative qRT-PCR, duck β-actin was used as a reference gene. The relative expression level of each gene was calculated by the 2^−ΔΔCt^ method [60], and was normalized to that of β-actin. Changes in gene expression were calculated by the *t*-test. Differences were considered statistically significant when *p* < 0.05.

## 5. Conclusions

In conclusion, our study revealed the transcriptome profiles of DTMUV-infected duck brain. We identified several pathways and genes related to the nervous system. RIG-I-like receptors (DHX58, IFIH1) and Toll-like receptors (TLR2 and TLR3) were activated, inducing the expression of interferon stimulated genes and antigen-processing and -presenting genes in the brain. The current study provides a comprehensive understanding of host–pathogen and neuro-immune interactions following DTMUV infection, which might provide a basis for the control of DTMUV and other neurovirulent flaviruses.

## Figures and Tables

**Table 1:** Statistics of sequencing reads.

Samples	Raw Reads	Clean Reads ^1^	Clean Reads Ratio ^2^	Total Mapped Reads^3^	Total Mapped Ratio ^4^
12 h-Control	52,151,546	51,155,090	98.09%	39,377,403	76.98%
24 h-Control	50,060,388	49,233,950	98.35%	38,039,893	77.26%
48 h-Control	52,859,536	51,873,096	98.13%	39,886,284	76.89%
12 h-Infected	58,372,706	57,108,946	97.84%	43,294,720	75.81%
24 h-Infected	40,835,734	40,030,410	98.03%	30,830,799	77.02%
48 h-Infected	50,284,812	49,197,138	97.84%	37,552,725	76.33%

^1^ Clean reads: Raw reads filtering out ribosomal RNA, adapters, and low quality reads; ^2^ Clean reads ratio: (Clean reads/Raw reads) %; ^3^ Total mapped reads: Reads mapped to chicken genomes; ^4^ Total mapped ratio: (Total mapped reads/Clean reads) %.

**Table 2:** KEGG pathways related to immune responses during duck Tembusu virus (DTMUV) infection.

Time Point	Pathway	*p*-Value	Up-Regulated	Down-Regulated
12 h	RIG-I-like receptor signaling pathway	3.43 × 10^−4^	DHX58, IRF7, TRIM25	—
24 h	RIG-I-like receptor signaling pathway	3.64 × 10^−4^	DHX58, IRF7, TRIM25, IFIH1	—
	Toll-like receptor signaling pathway	2.57 × 10^−5^	TLR2, TLR3, STAT1, IRF7, LOC101791385, LOC101791569	—
	Chemokine signaling pathway	1.38 × 10^−2^	STAT1, PAK1, LOC101791385, LOC101791569	—
	Antigen processing and presentation	2.93 × 10^−4^	TAP1, TAP2, LOC101797502, LOC101790497	—
	Natural killer cell mediated cytotoxicity	3.31 × 10^−3^	PAK1, BID, LOC101797502, LOC101790497	—
	Complement and coagulation cascades	1.18 × 10^−2^	C1S, C1R	LOC101801552
48 h	RIG-I-like receptor signaling pathway	1.03 × 10^−3^	DHX58, IRF7, TRIM25	—
	Toll-like receptor signaling pathway	1.84 × 10^−3^	STAT1, IRF7	TLR2
	JAK-STAT signaling pathway	9.03 × 10^−4^	STAT1, LOC101793812	LOC101803793, LOC101789923
	Antigen processing and presentation	9.13 × 10^−3^	B2M	LOC101797502

## Supplementary Materials

Supplementary materials can be found at https://www.mdpi.com/1422-0067/21/7/2402/s1.
