# Supplementary material for: Transcriptome Analysis Reveals the Neuro-Immune Interactions in Duck Tembusu Virus-Infected Brain
Source: Int J Mol Sci. 2020 Mar 31;21(7):2402. doi: 10.3390/ijms21072402 (PMC7177238; doi:10.3390/ijms21072402)
Supplement: Supplementary file 1 [file ijms-21-02402-s001.zip › Table S3 primers qRT-PCR.docx]

Table S3 Primers for qRT-PCR

| Names | Primers |
| --- | --- |
| TMUV-E-F | CCGCCAGATTCGTTATAAC |
| TMUV-E-R | CATGGTAAGTTGAGATCATG |
| LOC101796523-F | GGAGATGATAACTTGCCAGG |
| LOC101796523-R | GTAGTAGACCCTTGGGACTGTTC |
| LOC101791000-F | AGGAATCATTGGTGTCAAGGGT |
| LOC101791000-R | ACATTCCACAACTCTATCAGGT |
| IFIT5-F | TAGACACTGTTGTTATGGCAC |
| IFIT5-R | GTGCCTTGTCCACTTTCCCTTT |
| IFI35-F | CATCGCTAGAGGGCAAGT |
| IFI35-R | TGGAACTGGATGTTAGTGACCT |
| IRF7-F | CGCCACCCGCCTGAAGAAGT |
| IRF7-R | CTGCCCGAAGCAGAGGAAGAT |
| USP18-F | TATCTGACTCTCTGGAACTTGGTAA |
| USP18-R | CTGGGAGTGGAAGGGTTA |
| TRIM25-F | CAACTGCGGCTAATACAA |
| TRIM25-R | GAAATCCAGGGTGATGTTA |
| RSAD2-F | GCGTGGTCAAGGAAAGAA |
| RSAD2-R | TGAAAGCAACAGCATACTC |
| β-actin-F | GGTATCGGCAGCAGTCTTA |
| β-actin-R | TTCACAGAGGCGAGTAACTT |
